# Pharmacy Students’ Perspectives on Integrating Generative AI into Pharmacy Education

**DOI:** 10.3390/pharmacy13060183

**Published:** 2025-12-15

**Authors:** Kaitlin M. Alexander, Eli O. Jorgensen, Casey Rowe, Khoa Nguyen

**Affiliations:** 1 Department of Pharmacy Education and Practice, University of Florida College of Pharmacy, Gainesville, FL 32610, USA; casey.rowe@ufl.edu; 2 Department of Pharmacy Services, UF Health Shands Hospital, Gainesville, FL 32608, USA; eli.jorgensen@ufhealth.org; 3 Department of Pharmacotherapy and Translational Research, University of Florida College of Pharmacy, Gainesville, FL 32610, USA; khoanguyen@cop.ufl.edu

**Keywords:** artificial intelligence, pharmacy education, pharmacy, pharmacy student

## Abstract

**Objective:** This study aims to evaluate pharmacy students’ perceptions regarding the integration of generative artificial intelligence (GenAI) into pharmacy curricula, providing evidence to inform future curriculum development. **Methods:** A cross-sectional survey of Doctor of Pharmacy (PharmD) students at a single U.S. College of Pharmacy was conducted in April 2025. Students from all four professional years (P1–P4) were invited to participate. The 10-item survey assessed four domains: (1) General GenAI Use, (2) Knowledge and Experience with GenAI Tools, (3) Learning Preferences with GenAI, and (4) Perspectives on GenAI in the curriculum. **Results:** A total of 110 students responded (response rate = 12.4%). Most were P1 students (56/110, 50.9%). Many reported using GenAI tools for personal (65/110, 59.1%) and school-related purposes (64/110, 58.1%) sometimes, often, or frequently. ChatGPT was the most used tool. While 40% (40/99) agreed or strongly agreed that GenAI could enhance their learning, 62.6% (62/99) preferred traditional teaching methods. Open-ended responses (*n* = 25) reflected a mix of positive, neutral, and negative views on GenAI in education. **Conclusions:** Many pharmacy students in this cohort reported using GenAI tools and demonstrated a basic understanding of GenAI functions, yet students also reported that they preferred traditional learning methods and expressed mixed views on incorporating GenAI into teaching. These findings provide valuable insights for faculty and schools of pharmacy as they develop strategies to integrate GenAI into pharmacy education.

## 1. Introduction

Artificial intelligence (AI) has the potential to revolutionize healthcare and pharmacy practice by optimizing medication management, enhancing pharmacy operations, and accelerating drug discovery [1,2]. With rapid advancements in generative artificial intelligence (GenAI) technology, it is important for current pharmacy students to acquire knowledge and understand GenAI to be adequately prepared for their future careers [3]. AI-related clinical competencies for healthcare professionals include foundational knowledge of AI, understanding its ethical implications, recognizing clinical impacts, evaluating AI tools, analyzing workflow integration, and committing to lifelong learning in the context of AI in healthcare [4]. Pharmacy curricula should consider how to develop these skills to enable students to effectively use AI in clinical practice, as understanding AI ethics and ensuring the ability to critically evaluate AI tools will likely be important for their future careers.

Given that existing pharmacy curricula were developed prior to the widespread access to GenAI technologies, many colleges of pharmacy may not have fully implemented a plan to educate students about AI. In addition, U.S. pharmacy accreditation standards do not directly address the need to incorporate AI into curricula, rather they reference digital health within the knowledge domain of curriculum standard 2 [5]. As GenAI becomes increasingly accessible, some pharmacy faculty have begun integrating it into their teaching by incorporating GenAI in drug information assignments, using it to support communication skills development, and applying it to teach concepts related to drug discovery [3,6,7,8,9,10]. However, these efforts are often initiated by individual instructors rather than systematically incorporated into the curriculum and therefore may not comprehensively address student AI competencies that need to be developed [6,7,8,9,10].

Previous surveys of pharmacy learners have shown that students are using GenAI technologies for both personal and academic purposes, with varying levels of enthusiasm and caution towards GenAI integration [11,12,13,14]. Additionally, students’ perceptions on how and where to integrate GenAI teaching into their training are underexplored [12,13,15]. Understanding students’ experiences, expectations, and concerns surrounding AI can guide faculty in developing AI content that aligns with learner needs.

At our institution, access to GenAI tools has expanded with the introduction of university-supported GenAI platforms, which may influence student use. Additionally, faculty have increasingly incorporated AI-related content into teaching and implemented activities utilizing GenAI, raising important questions about how students perceive these changes. Finally, college policies on GenAI use have also evolved, updating from a strictly prohibited approach to one that permits GenAI use on assignments and activities with faculty approval. Therefore, the purpose of this study is to evaluate pharmacy students’ perceptions regarding the integration of GenAI into the pharmacy curriculum, providing evidence to inform future curriculum development.

## 2. Methods

This was a cross-sectional survey of pharmacy students at a single U.S. college of pharmacy at a large, public university and health science center. The university supports access to a variety of GenAI tools for faculty and students, including NaviGator Chat, a custom-built, comprehensive, self-service AI platform hosted by the university (University of Florida, Gainesville, FL, USA), and Microsoft Copilot (Microsoft 2025 version, Redmon, WA, USA).

All students within the Doctor of Pharmacy (PharmD) program, from the first year to the fourth year (P1–P4), were invited to participate. The survey was conducted in April 2025. It was distributed via email, and through an announcement in the program’s learning management system, Canvas. At the time of the survey, students had access to university-supported GenAI tools; however, no formal training on the use of these tools had been provided. Some faculty had begun incorporating GenAI tools into teaching and learning activities in P1–P3 didactic courses and P4 experiential learning. The survey was created by a research team comprised of three full-time faculty, an adjunct faculty, and a fourth-year PharmD student. The research team reviewed potential questions for inclusion, determined feasibility and ease of response, and identified errors for the survey. The college’s AI Task Force, a multidisciplinary group of faculty, staff, and students leading AI education at the college, reviewed the survey and provided feedback. Feedback was then incorporated, and final survey questions were agreed upon by the research team.

The survey was developed and disseminated using Qualtrics^®^ (2025 webpage version, Provo, UT, USA), an online survey creation and distribution software. The survey consisted of 10 items that evaluated four general domains: (1) General GenAI Use, (2) Knowledge and Experience with GenAI Tools, (3) Learning Preferences with GenAI, and (4) Student Perspectives on GenAI at the college. First, General GenAI Use was assessed through questions about usage frequency and specific GenAI tools utilized. Second, Knowledge and Experience with GenAI Tools was measured using five-point Likert scales (ranging from Strongly Disagree to Strongly Agree) addressing understanding, application, knowledge, and evaluation capabilities. Third, Learning Preferences with GenAI employed the same five-point scale to assess students’ preferences for learning, training, and GenAI integration. Finally, Student Perspectives on GenAI at the college were captured through a combination of free-response questions, five-point frequency scales (Never to Frequently), and ranking items.

Participation in the survey was voluntary and estimated to take 5–10 min. The survey was anonymous, and demographic information was not collected. Participants could exit the survey at any time and partially completed surveys were included. Responses to completed items were analyzed.

Responses were analyzed using descriptive statistics performed in Microsoft Excel (Microsoft 365 2025 version, Redmon, WA, USA) and reported as an N (%). Likert scale responses were converted to numerical values (1 = Strongly Disagree to 5 = Strongly Agree) and summarized using median and interquartile range (IQR). Open-ended student comments were reviewed by the research team and summarized descriptively. This study was approved by the university’s institutional review board.

## 3. Results

A total of 110 students out of the 888 total students enrolled responded to the survey (12.4% response rate). Fewer students provided responses on their Learning Preferences with GenAI Tools and Student Perspectives on GenAI at the college (*n* = 99; 11.2% response rate). Most respondents were in their first year of the professional PharmD program (56/110, 50.9%), followed by fourth-year students at 19.1% (21/110), second-year students at 17.3% (19/110), and third-year students at 12.7% (14/110).

### 3.1. General GenAI Use 

General GenAI Use responses are reported in Table 1. Students showed emerging adoption of GenAI tools with many reporting using GenAI tools for personal (59.1%) or school-related purposes (58.1%) sometimes, often, or frequently. Regarding AI tool utilization,14 tools were reported by the respondents (Table 1). ChatGPT, NaviGator Chat, and Gemini were the most frequently utilized. Twenty students (18.2%) reported no previous experience with GenAI.

### 3.2. Knowledge and Experience with GenAI Tools 

Responses regarding Knowledge and Experience with GenAI Tools are reported in Table 2**.** Based on a six-item assessment, students generally reported demonstrable knowledge and experience with GenAI tools. Sixty percent of students (66/110) either agreed or strongly agreed that they understand the basic concepts of AI. Similarly, most students responded that they are knowledgeable about AI ethics and policies in education (76/110, 69.1%), able to critically evaluate GenAI tools for effectiveness (63/110, 57.3%), and able to critically evaluate GenAI tools for ethical considerations (62/110, 56.4%). Most students appeared to be hesitant about GenAI reliability, with only 22.7% (25/110) reporting that they trust GenAI tools for academic purposes.

### 3.3. Learning Preferences with GenAI 

Responses regarding Learning Preferences with GenAI are reported in Table 3. Based on six statements, the results were generally mixed regarding their learning preferences. Roughly forty percent of students (40/99, 40.4%) reported that GenAI tools would enhance their learning, while others responded that they still prefer traditional methods of teaching over AI (62/99, 62.6%). Despite this, some students responded that they would like to learn more about how faculty use AI in their practices (50/99, 50.1%) and that they would benefit from clearer guidelines on the appropriate and ethical use (51/99, 51.5%).

### 3.4. Student Perspectives on GenAI

Students reported that they perceive that faculty incorporate AI tools into their teaching only sometimes (51/99, 51.5%) or rarely (29/99, 29.3%). When asked in which course types they have experienced AI integration, patient care-focused courses were reported most frequently. Students were also asked which courses they believed that AI integration would be the most beneficial. Students were mixed in their views of the courses where AI could be integrated, with patient care-focused courses being reported most often followed by orientation sessions or workshops. Twenty-four students (24.2%) responded “none”.

Students were also permitted to leave open-ended comments regarding their perceptions of integrating GenAI into the curriculum. Twenty-five students provided comments, and they were a mix of positive, neutral, and negative viewpoints on GenAI in education. Students shared that incorporating GenAI was perceived as offering additional opportunities for practice, enhancing their learning and helping prepare them for future pharmacy practice. Some learners also expressed that GenAI use should remain optional and serve as a supplement to faculty instruction. Finally, some students noted that GenAI output can be unreliable, may detract from their education, and could encourage ‘laziness’.

## 4. Discussion

The current study reveals that pharmacy students in this cohort hold a cautiously optimistic attitude towards GenAI in education. While many students are already using GenAI tools for both personal and academic purposes and report a basic understanding of GenAI tools, there remains a notable hesitation about AI’s integration into their learning. Results suggested that this reluctance may be related to a preference for traditional educational methods, indicating that they value the human elements of instruction and are not yet fully confident in AI’s role in education. Students expressed interest in learning more about how faculty use AI in practice, suggesting that incorporating examples of AI applications in pharmacy could be an effective way to introduce students to AI.

Previous survey studies from 2023 reported that approximately 20–30% of pharmacy students are using GenAI for academic purposes [11,13]. However, our survey indicates a notable rise in GenAI adoption, with 81.8% of students reporting that they have used GenAI tools at least once for school-related tasks. This rise is expected, given the growing accessibility and integration of GenAI tools into everyday life and the previous surveys were conducted shortly after the launch of OpenAI’s Chat GPT. Since then, university-supported GenAI tools have become more widely available, and we’ve seen a rise in faculty incorporating AI into classroom activities, which also likely influenced student usage.

Despite the increase in GenAI use, some students expressed a lack of trust in GenAI for academic tasks, which may also demonstrate a rise in awareness of GenAI limitations [11]. This presents an opportunity to provide training on critically evaluating GenAI outputs and applying the AI-generated information appropriately in academic and professional contexts. While a degree of skepticism is warranted (since all information should be verified) we also want students to feel confident using GenAI tools responsibly, given their widespread adoption.

The rapid growth in GenAI adoption among pharmacy students has important implications for pharmacy educators. Overall, students self-reported high confidence in their understanding and knowledge of GenAI tools, reflecting increased familiarity and comfort with GenAI technologies. As student use of GenAI becomes more prevalent, it is essential to foster a culture of transparency and responsible GenAI use within academic settings. Educators should develop clear course policies that guide responsible GenAI use. These policies should clearly define acceptable use of GenAI and provide guidance to learners on how to disclose its use (e.g., specifying the AI tool utilized and describing how it was applied in completing an assignment or activity). Policies should also outline situations where GenAI use is prohibited, such as during proctored assessments, exams, or quizzes, in accordance with course requirements [16]. Finally, policies should emphasize the importance of maintaining both patient and student confidentiality and specify the consequences for inappropriate use [17].

Importantly, many students indicated that they would benefit from additional training on how to use GenAI tools in education and clearer guidance on their appropriate and ethical use. These findings have significantly influenced our work at the college. In response, we developed plans to implement an AI seminar for all students in the didactic curriculum (P1–P3). The seminar will cover key topics such as the impact of AI on pharmacy practice, foundational AI concepts, guidance on using GenAI tools for learning, a review of college policies on GenAI use, and training on institutionally available GenAI tools. Another potential benefit is that this training may help streamline future teaching, reducing the need to revisit AI basics repeatedly later in the curriculum.

There is also a growing need for instructors to consider redesigning assessments, particularly for tasks that can be easily completed using GenAI [18]. Traditional assessments that rely heavily on basic recall may no longer effectively measure student learning, as these are areas where GenAI performs well. Instructors may consider a shift toward authentic assessments that emphasize higher order thinking skills, such as analysis and evaluation [19]. When designing assessments, faculty can also evaluate whether allowing learners to use GenAI would compromise their learning. If GenAI use does not undermine the learning objective, there may be opportunities to integrate GenAI into assignments in a structured and responsible manner. Conversely, if GenAI use would diminish learning, assessments should be adapted by either moving them into a proctored environment where AI use can be monitored or by implementing alternative assessment formats that emphasize and assess critical thinking and problem-solving skills rather than basic recall [3,18,19,20].

Many students in the survey reported using OpenAI’s ChatGPT, which may highlight important considerations around tool selection and training. Students may default to general-purpose chatbots rather than choosing secure, university-supported platforms or research-specific tools. Some students may have been unaware of the university-supported GenAI platform available to them (Navigator Chat) and the advantages of using this tool. To address this, training on the availability and benefits of institutionally supported GenAI tools is planned. Additionally, introducing students to research-specific or healthcare-focused GenAI tools could help guide students in using AI effectively for specialized tasks Finally, it is essential to educate students on digital ethics and data protection, emphasizing that sensitive academic or patient information should never be entered into open-access large language models.

When assessing students’ learning preferences related to GenAI, the responses were mixed regarding whether they agreed that AI would enhance their learning. A significant number of students preferred traditional educational methods and disagreed with incorporating more AI tools into teaching. However, students expressed curiosity about how faculty use AI in pharmacy practice. Demonstrating real-world applications of AI in pharmacy and healthcare may help students better understand AI’s relevance and offer a practical approach to curricular integration [4,21]. Another important consideration is that patients are also using GenAI tools to advocate for their health. Therefore, future pharmacists must be prepared to address this patient use. Faculty development offers an opportunity to encourage instructors to thoughtfully revise activities to incorporate GenAI and highlight its impact within their disciplines. These efforts can help address student hesitation and reservations about GenAI adoption by demonstrating its relevance to pharmacy practice and their future careers.

Several limitations should be considered when interpreting the findings of this study. First, the data were collected from a single U.S. college of pharmacy that provides students and faculty with access to a variety of GenAI tools, which may limit the generalizability of the results to other institutions without these resources, as this access may have contributed to increased adoption and use of GenAI among students. Additionally, the response rate was low and varied across student cohorts, with a higher proportion of P1 respondents compared to upperclassmen. This imbalance limits the representativeness of the findings, as P1 students may differ from students in later years in terms of attitudes and experiences with GenAI. P1 students may be more receptive to new technologies but have limited exposure to the pharmacy curriculum and minimal experience applying GenAI in the context of pharmacy. In addition, their attitudes could change (becoming more positive or negative) as the students gain additional experience in the curriculum. There is also potential for response bias, such as students underreporting or hesitating to disclose their use of GenAI. However, the anonymous nature of the survey and its administration outside of any course context likely encouraged honest responses. Finally, the survey did not collect demographic information, such as age, which could influence students’ perceptions and use of GenAI.

This study contributes to the growing body of literature on student use and perceptions of GenAI in pharmacy education by providing updated insights and data. Future research should further explore specific areas of teaching and learning where students perceive greater or lesser value in the use of GenAI tools. As GenAI use increases among learners, pharmacy programs must prepare students to engage with these technologies responsibly and effectively.

## 5. Conclusions

In conclusion, many pharmacy students in this cohort reported using GenAI tools and demonstrated a basic understanding of their functions. Despite this, they expressed a preference for traditional learning methods and offered mixed opinions on whether faculty should incorporate more GenAI tools into teaching. However, students showed interest in learning about practical examples of AI applications in pharmacy practice. These findings provide valuable insights for faculty and schools of pharmacy as they develop strategies to integrate GenAI into pharmacy education.

## Figures and Tables

**Table 1:** Frequency of Generative AI Use Among Pharmacy Students (*n* = 110).

Survey Item	Participant’s Response, *n* (%)					
	Never	Rarely	Sometimes		Often	Frequently
How often do you use AI/LLM tools for personal purposes?	20 (18.2)	25 (22.7)	32 (29.1)		19 (17.3)	14 (12.7)
How often do you use AI/LLM tools for school-related purposes?	20 (18.2)	26 (23.6)	33 (30.0)		16 (14.5)	15 (13.6)
**Generative AI Tools Used by Pharmacy Students, *n* (%)**						
Which AI tools have you used?	ChatGPT			98 (89)		
	NaviGator Chat			32 (29)		
	Gemini			18 (16)		
	Microsoft Copilot			9 (8)		
	Google Notebook LM			7 (6)		
	Perplexity			6 (5)		
	None			4 (4)		
	ClaudeAI			3 (3)		
	Other			7 (6)		

Key: LLM = large language model, AI = artificial intelligence.

**Table 2:** Knowledge and Experience with AI Tools Among Pharmacy Students (*n* = 110).

Survey Item	Participant’s Response, *n* (%)					Median (IQR)
	Strongly Disagree (1)	Disagree (2)	Neither Agree nor Disagree (3)	Agree (4)	Strongly Agree (5)	
I understand the basic concepts of AI	3 (2.7)	5 (4.5)	36 (32.7)	33 (30.0)	33 (30.0)	4 (3–5)
I can effectively apply AI tools in my coursework and studies	9 (8.2)	14 (12.7)	36 (32.7)	24 (21.8)	27 (24.5)	3 (3–4)
I am knowledgeable about AI ethics and policies in education	4 (3.6)	5 (4.5)	25 (22.7)	29 (26.4)	47 (42.7)	4 (3–5)
I am able to critically evaluate AI tools for their effectiveness	5 (4.5)	9 (8.2)	33 (30.0)	26 (23.6)	37 (33.6)	4 (3–5)
I trust LLM tools for academic purposes	26 (23.6)	20 (18.2)	39 (35.5)	13 (11.8)	12 (10.9)	3 (2–3)
I am able to critically evaluate AI tools for their ethical considerations	7 (6.4)	10 (9.1)	29 (26.4)	29 (26.4)	33 (30.0)	4 (3–5)

Key: LLM = large language model, AI = artificial intelligence, IQR = interquartile range, 1 = strongly disagree to 5 = strongly agree.

**Table 3:** Learning Preferences with AI Tools Among Pharmacy Students (*n* = 99).

Survey Item	Participant’s Response, *n* (%)					Median (IQR)
	Strongly Disagree (1)	Disagree (2)	Neither Agree nor Disagree (3)	Agree (4)	Strongly Agree (5)	
AI tools would enhance my learning	21 (21.2)	14 (14.1)	24 (24.2)	28 (28.3)	12 (12.1)	3 (2–4)
Faculty should incorporate more AI tools into their teaching	28 (28.3)	15 (15.2)	29 (29.3)	19 (19.2)	8 (8.1)	3 (1–4)
I prefer traditional methods over AI tools in my learning	0 (0)	10 (10.1)	27 (27.3)	18 (18.2)	44 (44.4)	4 (3–5)
I would like to learn more about how faculty use AI in their practices	15 (15.2)	14 (14.1)	20 (20.2)	24 (24.2)	26 (26.3)	4 (2–5)
I would benefit from training on how to use AI tools in education	19 (19.2)	13 (13.1)	25 (25.3)	21 (21.2)	21 (21.2)	3 (2–4)
I would benefit from clearer guidelines on the appropriate and ethical use of AI	13 (13.1)	10 (10.1)	25 (25.3)	23 (23.2)	28 (28.3)	4 (3–5)

Key: AI = artificial intelligence, 1 = strongly disagree to 5 = strongly agree.

## Data Availability

The original contributions presented in this study are included in the article. Further inquiries can be directed to the corresponding author.
